# A Foundational “Survival Guide” Overview of Sports-Related Head Injuries

**DOI:** 10.7759/cureus.11636

**Published:** 2020-11-22

**Authors:** Frank De Stefano, Brian Fiani, Tim Mayo

**Affiliations:** 1 Neuroscience, Kansas City University of Medicine and Biosciences, Kansas City, USA; 2 Neurosurgery, Desert Regional Medical Center, Palm Springs, USA

**Keywords:** traumatic brain injury, brain trauma injury, epidural hematoma, acute subdural hematoma, brain concussion, traumatic subarachnoid hemorrhage, diffuse axonal injury

## Abstract

Around 3.8 million traumatic brain injuries (TBI) occur every year from athletic participation. The signs and symptoms of each specific head injury can be difficult to delineate. Further, treatment for each injury varies significantly. While most sports-related head injuries are not life-threatening, prompt recognition of acute head injury with expedited care leads to better outcomes. Current medical education lacks in awareness of common sports-related head injuries and the acute management of these injuries. Due to this, a literature review was originally crafted to provide medical students with a brief education in the recognition, diagnosis, and acute management of sports-related head injuries. The objective is to provide a “survival guide” style of reference for medical students, but may also be useful for all primary care providers, first line responders, and athletic trainers. Current guidelines and primary studies were investigated to delineate common head injuries and their associated medical management. With this data, we developed a brief, overview regarding common head injuries that occur in sport-related activities. In addition to listing the most common brain injuries, we elaborate on how to develop acute care plans specific to each type of injury. The treatment plans could be enhanced via stratification into sex and age subcategories, as well as through studies including data regarding long-term observation.

## Introduction and background

An estimated 38 million children and 170 million adults annually participate in organized sports [1]. Organized sports provide a wide range of physical and mental health benefits and lower the risk of chronic, lifestyle-induced conditions like cardiovascular disease and metabolic disorders [2,3]. Competitive play in these activities inevitably leads to injuries. Head trauma resulting in brain injury is a significant issue in many contact and non-contact sports [4,5]. Proper neurosurgical care is critical in the acute management of these injuries to reduce or prevent long term damage. In this review, we will address the acute management of head injuries and different approaches for their management. While it is widely known that contact sports have significant risk for head injuries, many non-contact sports have similar or even higher rates of head injury, such as soccer, basketball, and cycling [6]. Although most head injuries that occur during sport are not life-threatening, it is critical to be aware of possible brain injuries and their management to care for athletes. It is gravely important that medical students, first-line responders, athletic trainers, and primary care providers be equipped with the knowledge of traumatic brain injury and for those individuals we provide this “survival guide” style reference as a brief foundational overview of traumatic brain injury.

## Review

Epidural hematoma

Epidural hematoma (EDH) is a rare, but serious, life-threatening injury seen in sports-related head injury. The pathophysiology behind this injury is pooling of blood in the epidural space between the skull and dural layer of the brain. The source of bleeding is commonly due to rupture of the middle meningeal artery or vein from temporal bone fracture. Seventy-five percent of patients with EDH have skull fracture at diagnosis [7]. The expanding blood volume increases intracranial pressure resulting in brain herniation. This sequence ultimately results in brain damage, coma, and death. A common presentation of an athlete seen with EDH is the so-called “lucid interval”. These patients have a transient loss of consciousness with recovery followed by a slow increase in severity of clinical symptoms over time. The lucid interval is important to recognize in patients with epidural hematoma for early treatment. However, a systematic review found that this clinical manifestation occurs in 47% of all cases [8].

Diagnosis and Treatment of Epidural Hematoma

Non-contrast enhanced computed tomography (CT) is the gold standard for initial evaluation of EDH, as well as many other suspected intracranial lesions, due to its speed, cost, and availability [9]. Magnetic resonance imaging (MRI) performs at higher sensitivity than CT in detection of intracranial lesions and should be utilized in circumstances of equivocal findings on CT [10]. The key signature on brain imaging is the “lens” shaped hematoma due to the blood volume confined by the dural attachments to the cranial bone sutures.

Hematoma volume is the critical factor in the decision-making model for the acute care of athletes with EDH and can be evaluated using the ABC/2 mathematical formula. ABC/2 is a calculation based on brain imaging to assess for intracranial hematoma volume. However, caution should be taken for lesions that are grossly lobular, irregular, or hypo-attenuated. The ABC/2 score is calculated as followed:

A = Greatest diameter of hemorrhage on CT slice of largest area 

B = Greatest diameter 90 degrees to “A” measurement 

C = Number of CT slices involved with lesion multiplied by CT slice thickness

Each CT slice should be compared with the slice utilized in factors A and B. Calculation should be area of hemorrhage on slice of interest in comparison slice of largest hemorrhage: >75% = one slice, 25-75% = half slice, <25% = slice should not be counted. A meta-analysis conducted to determine accuracy of volume estimation found the ABC/2 calculation to be clinically acceptable. The study found the calculation method did not significantly deviate from manual planimetry [11].

The clinical algorithm for management of EDH deviates from the decision of surgical intervention vs. clinical observation. Indications for surgery are based on clinical presentation and imaging findings. Current guidelines state patients with hematoma volume on imaging found to be greater than 30 cm^3^ should be surgically evaluated regardless of clinical presentation. Patients with a Glasgow Coma Scale (GCS) < 9 and anisocoria are recommended for surgical evaluation. Patients presenting with hematoma volume less than 30 cm^3^, midline shift of less than 5 mm, clot thickness < 15 mm, and GCS > 8 can be ruled out for surgical evaluation and managed non-operatively [12].

The overwhelming majority of EDH cases undergo surgical intervention. As imaging has advanced, more patients have been managed conservatively without surgical intervention [13]. Great attention must be given to monitor for potential expansion of the hematoma post-injury. A retrospective study found that 23% of patients treated non-operatively for EDH reported increased expansion of the hematoma early in the clinical course at an average of eight hours post-injury [14]. While the literature has not concluded on a definitive protocol, this evidence suggests close monitoring of the patient for neurologic function with serial imaging is warranted. 

Subdural hematoma

Acute subdural hematoma (SDH) is the most common type of intracranial bleeding in sports-related head injuries and is also the leading cause of death or severe disability in sports-related head injuries [15]. SDH is a traumatic brain injury due to rupture of bridging veins crossing the subdural space. This hemorrhagic event accumulates between the meningeal dura and arachnoid mater of the brain. Like EDH, this expanding hematoma increases pressure in the cranial cavity that can result in ischemia, irreversible damage, and brain herniation. Prognostic outcomes for SDH are poor. Overall mortality for patients requiring surgical intervention is 60% with worse outcomes in patients presenting with focal neurologic deficits at time of medical attention [16]. The athlete diagnosed with SDH likely arose from forces applied to the skull from contact with the ground. Unlike the intracranial anatomy of elderly patients, athletes tend to have less subdural space from less brain atrophy. This results in elevated intracranial pressures in a shorter amount of time. SDH in athletes also presents more often with concomitant brain parenchyma contusions due to coup and counter-coup injuries, worsening clinical outcomes [17].

Diagnosis and Treatment of Subdural Hematoma

Like EDH, non-contrast enhanced CT is first choice for imaging of suspected acute SDH. The classic imaging signature for acute SDH is the “crescent” shaped hematoma not confined to suture limitations in comparison to EDH. Common locations for this type of injury include the middle cranial fossa and frontoparietal regions. In the acute phase, the hematoma appears homogenous and hyperdense but reduction in intensity is common as time from injury progresses [18].

Not all patients presenting with SDH require surgery. Due to the acute nature of trauma in athletes, there should be no delay in determining if surgical intervention is appropriate. Factors determining surgical evaluation should be made on imaging findings and the patient’s neurologic status from time of injury to medical attention. Like EDH, the ABC/2 calculation is a validated approach to determine the volume of subdural hematoma [19]. Current guidelines to indicate surgical evaluation of patients with acute SDH include all patients with hematoma thickness > 10 mm or midline shift > 5 mm. This recommendation is for all patients regardless of GCS score. Regarding neurologic status, any patient that has a GCS decrease greater than 2 from injury to medical attention, along with any pupil abnormalities, should be surgically evaluated. This is regardless of imaging findings. Candidates eligible for non-operative care of acute SDH require clot thickness < 10 mm, midline shift < 5 mm, no pupillary abnormalities, and no intracranial hypertension. For patients treated non-operatively with GCS < 9 at presentation, surgical evaluation is indicated if intracranial pressure (ICP) is persistently elevated > 20 mm Hg after medical therapy [16]. Prompt surgical intervention is necessary as soon as the decision is made for surgical evaluation. One study found that mortality rates for patients treated surgically within four hours of injury or longer than four hours were 30% and 90%, respectively [20].

Surgical Management of EDH and SDH

The mainstay of surgical management for acute EDH and SDH is evacuation of the hematoma to relieve intracranial pressure via craniotomy or decompressive craniectomy. The literature suggests no significant difference in clinical outcomes when deciding on craniotomy or craniectomy. This decision should be made on a case-by-case basis taking into account other factors of the patient [21]. The available literature concludes that patients with intracranial bleeding requiring surgical evaluation should be operated on as soon as possible to prevent adverse outcomes [22]. 

Diffuse axonal injury

Diffuse axonal injury (DAI) is a life-threatening traumatic brain injury (TBI) associated with poor outcomes. The forces involved in this kind of injury require strong acceleration-deceleration commonly seen with high-frequency shaking or strong rotation forces placed on the skull. These forces result in the tearing of axons most notably at areas of high density in the brain, notably the gray-white junction [23]. It is important to note that the primary injury, as well as secondary injury, play an important role in the clinical outcomes of DAI. Primary injury disrupts cytoskeleton transport needed to carry out axonal function. This damage eventually results in the swelling that causes dissection of the axon. The so-called “retraction ball” defines the moment when the proximal portion of the axon retracts back to the neuron body. This phenomenon is pathognomonic for DAI [24]. Primary injury involved with DAI initiates a cascade of neuroinflammation, metabolic dysregulation, and axonal death. DAI is one of the most common pathologic findings associated with TBI. Signs of DAI are present in 50% of athletes with severe head injury [25]. Increasing severity of DAI at diagnosis correlates with long term functional recovery and poorer outcomes [26].

Characteristics of DAI are now being recognized in many forms of TBI. Patients with possible DAI present in a wide range of neurologic statuses from mild confusion to a fully comatose state, with most patients presenting with GCS < 8. Diagnosis of DAI is made clinically in patients with GCS < 8 for greater than six hours [27]. Punctate hemorrhages in the gray-white matter junction and corpus callosum are indicated as a classic imaging finding of DAI. CT imaging has low utility in detection of DAI, as this finding is present only 10% of the time [28]. However, CT is indicated to rule out other forms of intracranial hemorrhage. MRI, specifically T2-weighted imaging, outperforms CT in sensitivity of detecting DAI. Newer imaging modalities, such as diffusion tensor imaging, have been shown to correlate with severity of neurologic deficit at presentation and outperform high-resolution MRI in detecting longitudinal changes in patients with DAI [29].

Direct therapies for treatment of DAI remain limited. Treatment of DAI is largely supportive and includes airway management, hemodynamic stabilization, and reducing intracranial pressure. Potential therapies in the future consist of stem cell therapy, calcineurin modulators, docosahexanoic acid, and recombinant erythropoietin that are currently being investigated [30].

Mild traumatic brain injury/concussions

Mild traumatic brain injury (mTBI) a traumatically induced brain injury that causes a decrease in brain function and at least one of the following:

1. Any period of loss of consciousness

2. Any report of amnesia before or after the inciting event

3. Any indication of altered mental status at the time of inciting event

4. Focal neurologic deficit(s), transient or non-transient

But does not exceed:

1. Loss of consciousness greater than 30 minutes

2. GCS < 13, 30 minutes post-injury

3. Amnesia greater than 24 hours [31]

mTBIs are estimated to make up 95% of all TBIs [32]. mTBIs have become a growing issue with advancements in detection and diagnosis and their associated long term outcomes. Sports, particularly contact sports, are seeing increased rates of mTBIs with growing evidence suggesting they have much more serious consequences than believed. One out of five high school football players will be diagnosed with mTBI every season [33]. A common misconception in the medical literature is the synonymous usage of concussion and mTBI. Concussion is a form of mTBI that presents without any structural abnormalities on imaging [34]. For either, the pathophysiology producing injury is due to mechanical forces that cause the brain to impact against the intracranial surface. This impact causes shearing and stretching of axonal neurons that initiates a cascade of pathological processes including neurotransmitter release, hypermetabolic state, and generation of free radicals. For this reason, evidence suggests mTBI/concussion may be a milder form of DAI [35].

Management of Concussion

Signs and symptoms of concussion vary depending on the individual injury. The most common symptoms include headache, dizziness, amnesia, and loss of consciousness. It is important to note that loss of consciousness occurs in only 10% of diagnosed concussions [36]. The most recent consensus suggests that someone should undergo further testing for concussion if they present with one of the following symptoms:

a) Symptoms - somatic (e.g. headache), cognitive (e.g. feeling like in a fog) and/or emotional symptoms (e.g. lability)

b) Physical signs (e.g. loss of consciousness, amnesia)

c) Behavioral changes (e.g. irritability)

d) Cognitive impairment (e.g. slowed reaction times)

e) Sleep disturbance (e.g. insomnia) [37]

Initial evaluation of a patient should include patient interview, full neurologic examination, and determination of the patient’s clinical status. Concussion is a clinical diagnosis. There are many assessment tools in practice today for diagnosis of concussion: Sports Concussion Assessment Tool (SCAT5), Child SCAT5, and Concussion Recognition Tool 5 (CRT5), Maddocks Questions, Balance Error Scoring System (BESS), and NFL Sideline Concussion Assessment Tool. It should be cautioned that no assessment tool has been validated for performance in diagnosis of concussion [37].

Imaging for mTBI and concussion is not indicated in all patients and is used solely to rule out other structural abnormalities, such as intracranial hemorrhage. Three criteria exist in practice today designed to help the provider determine which patients are indicated for further imaging: Canadian CT head rule (CCHR), the New Orleans Criteria (NOC), and the National Emergency X-Radiography Utilization Study II (NEXUS II). The American College of Emergency Physicians has endorsed their own criteria that indicate non-contrast head CT for patients with loss of consciousness or post-traumatic amnesia with one or more of the following: headache, vomiting, age greater than 60 years, drug or alcohol intoxication, deficits in short-term memory, physical evidence of trauma above the clavicle, post-traumatic seizure, GCS < 15, focal neurologic deficit, or coagulopathy [38].

Patients diagnosed with concussion should be closely monitored for 24 hours due to risk of neurologic deterioration or intracranial hemorrhage. Decision for in-hospital or home observation can be decided based on mental status, clinical symptoms, and neurologic function. Patients with any of the following are recommended for in-patient observation for at least 24 hours until symptoms improve: GCS < 15, abnormal findings on imaging, seizures, increased bleeding risk, neurologic deficit, or intractable vomiting [39]. Treatment of concussion focuses on minimizing physical activity and cognitive rest. Patients should gradually re-introduce themselves back into physical activity, work, or school. The efficacy of specific post-concussion rest protocols has not been studied to determine best practice [40]. No specific medications or procedures are available for the direct treatment of concussion. Pharmacologic therapy is recommended for symptoms associated with concussion including seizure prophylaxis and pain management for headaches.

Potential Complications of mTBI and Concussion

Symptoms of concussion typically resolve within one to two weeks of initial injury with conservative measures including cognitive rest and restricted physical activity. Post-concussion syndrome (PCS) is a prolonged set of symptoms occurring in a subset of patients diagnosed with mTBI and concussion, although PCS has been reported in more severe forms of TBI. Patients with PCS report multiple, persistent symptoms including headache, dizziness, insomnia, and fatigue months after initial injury. One study identified patients with PCS had on average three to four concussions previously with median duration of symptoms lasting six months [41]. It is important to note that management of initial mTBI or concussion should stress prevention of subsequent injuries in the future.

Prompt recognition and evaluation of mTBI or concussion are critical to prevent further brain injury or death. Second-Impact Syndrome (SIS) is a rare but potentially fatal condition resulting from failure to adequately recover from an initial head injury. During initial head injury, the brain is capable of altering metabolic and physiologic function to mitigate the resulting cerebral edema. In SIS, the brain is more vulnerable to mechanical stress from initial injury. The subsequent impact sets off a pathologic process called “malignant brain edema”, where the brain is unable to regulate intracranial pressure and cerebral perfusion. The resulting edema can result in brain herniation with resulting high morbidity and mortality. Juvenile head trauma syndrome is a disease reported in the literature with possible similar pathophysiology that is unique to children and adolescents [42]. Due to the rarity of their occurrence, more research is needed to fully elucidate both disease processes. Regardless, it is of critical importance to identify an athlete with potential head injury and remove them from play immediately. Complete brain trauma workup and safe reintegration back into play are necessary to prevent these potentially fatal complications.

Return to Play

Graduated Return to Play (GRP) (Table 1) is a set protocol developed by the Concussion in Sport Group (CISG) organization in which a player diagnosed with concussion progresses through graduated intervals to return to play [43]. The protocol is designed that an athlete remains on a certain level of physical and cognitive restriction for at least 24 hours or until asymptomatic on that level. Once asymptomatic, the athlete progresses to a higher level with less physical and cognitive restrictions. If any symptoms return or worsen, the athlete should move back to the previous step for an additional 24 hours. During the acute injury period, physical activity should be avoided.

**Table 1:** Graduated Return to Play (GRP) protocol by the Concussion in Sport Group (CISG)

	Rehabilitation stage		Functional exercise at each stage of rehabilitation	The objective of each stage	
	No activity	Complete physical and cognitive rest		Recovery	
	Light aerobic exercise	Walking, swimming, or stationary cycling keeping intensity,70% maximum predicted heart rate No resistance training		Increase heart rate	
	Sport-specific exercise	Skating drills in ice hockey, running drills in soccer. No head impact activities		Add movement e xercise	
	Non-contact training drills	Progression to more complex training drills, e.g. passing drills in football and ice hockey		Exercise, coordination, and cognitive load	
	Full contact practice	Following medical clearance participate in normal training activities		Restore confidence and assess functional skills by coaching staff	
	Return to play	Normal gameplay		-	
	The 5th International Conference on Concussion in Sport held in Zurich				

## Conclusions

Sports-related head injuries and resultant TBI continue to be an integral part of the education curriculum for medical personnel. Herein, we summarized the most common diagnoses and the related features of each type of head injury that could be acquired during athletic participation. Additionally, we have discussed the acute management and return to play guidelines. The overview of head injuries provided can serve as a brief foundational guide for medical students, first-line responders, primary care providers, athletic trainers, and even non-medical professionals such as athletic coaches. It should remain in the armamentarium of these medical practitioners or first-line responders. From the neurobiology and neurophysiology perspective, additional studies are needed to follow the recovery process of young athletes stratified by age and sex so that an individualized approach can be made to each patient. There is also a need for large prospective studies that monitor TBI patients in terms of the safety of having return to play with long-term follow-up.
